# Factors influencing engagement with patient‐directed and facilitated advance care planning interventions for patients with advanced cancer

**DOI:** 10.1002/cncr.70025

**Published:** 2025-08-07

**Authors:** Kimberly J. Rak, Renusha Indralingam, Aaron Richardson, Neha Dhole, Shane Belin, Monica C. Leon Cadavid, Yuxin Zhou, Georgeann Booth, Bernard Hammes, Betty Ferrell, Douglas B. White, Robert M. Arnold, Rebecca L. Sudore, Megan Crowley‐Matoka, Yael Schenker

**Affiliations:** ^1^ Department of Critical Care Medicine University of Pittsburgh Pittsburgh Pennsylvania USA; ^2^ Department of Family Medicine Brown University Providence Rhode Island USA; ^3^ Department of Medicine West Virginia School of Medicine Morgantown West Virginia USA; ^4^ Public Health Foundation of India New Delhi India; ^5^ Division of General Internal Medicine Section of Palliative Care and Medical Ethics University of Pittsburgh Pittsburgh Pennsylvania USA; ^6^ Palliative Research Center (PaRC) University of Pittsburgh Pittsburgh Pennsylvania USA; ^7^ Department of Nursing Zhongda Hospital School of Medicine Southeast University Nanjing China; ^8^ Florence Nightingale Faculty of Nursing Midwifery and Palliative Care Cicely Saunders Institute of Palliative Care Policy and Rehabilitation King's College London London UK; ^9^ School of Medicine University of Pittsburgh Pittsburgh Pennsylvania USA; ^10^ Respecting Choices La Crosse Wisconsin USA; ^11^ City of Hope Medical Center Los Angeles California USA; ^12^ Department of Geriatrics and Palliative Medicine Icahn School of Medicine at Mount Sinai New York New York USA; ^13^ Division of Geriatrics Department of Medicine University of California San Francisco California USA; ^14^ Feinberg School of Medicine Northwestern University Chicago Illinois USA; ^15^ UPMC Hillman Cancer Center Pittsburgh Pennsylvania USA

**Keywords:** advance care planning, comparative effectiveness research, patient engagement, patient‐centered care, qualitative research

## Abstract

**Background:**

Advance care planning (ACP) for patients with cancer is guideline recommended. However, there are significant challenges to engagement. Patient‐facing ACP programs have been developed to improve access and engagement. This study aimed to identify factors impacting engagement for patients with cancer generally, and specifically for two patient‐facing approaches.

**Methods:**

As part of a randomized trial comparing the effectiveness of facilitated versus patient‐directed ACP, interviews were conducted with patients with cancer who participated in the two interventions. A thematic analysis approach was used, iteratively reviewing transcripts to identify and group concepts into themes.

**Results:**

Between 2020 and 2023, 60 participants in the parent study were interviewed (31 facilitated and 29 patient‐directed). Several themes were identified that contributed to ACP engagement, including a realist attitude, increased skills from previous ACP, and motivational beliefs such as alleviating burden for loved ones. Features of ACP design that facilitated engagement included intellectualization and normalization of content. For facilitated ACP, patients valued the interpersonal exchange with the interventionist. For patient‐directed ACP, participants appreciated the ability to step away and come back to the website. Participants discussed how ACP needs to be an iterative process as people have varying capacity or motivation to engage at different timepoints.

**Conclusions:**

Many patients with cancer are motivated to engage in ACP to decrease burden on others and receive patient‐centered care. A constellation of factors interact as facilitators or barriers to engagement. Therefore, a one‐time, one‐size approach is not recommended; facilitating engagement may require flexible approaches offered at multiple time points.

## Abstract 2

A constellation of internal and external factors interact as facilitators or barriers to engagement with advance care planning. Therefore, a one‐time, one‐size approach is not recommended; facilitating engagement may require flexible approaches offered at multiple time points.

## INTRODUCTION

It has been more than a decade since the Institute of Medicine published recommendations to improve the quality of end‐of‐life care and align care received with patients’ values, goals, and preferences.^1^ One facet of these recommendations was to increase engagement with advance care planning (ACP), which includes components such as identifying one's medical preferences and a surrogate decision maker, communicating preferences to family and medical teams, and completing advance directives.^2^ For patients with cancer, ACP is a guideline‐recommended aspect of care and studies have found it improves end‐of‐life experiences.^3^, ^4^, ^5^, ^6^, ^7^, ^8^ However, there are significant gaps in ACP engagement for patients with cancer, including an overall lack of, late, and/or one‐time engagement.^6^, ^9^, ^10^

Patient‐facing programs have been developed to improve access and engagement with ACP. The Patient Centered and Efficacious Advance Care Planning in Cancer compare trial compared the effectiveness of two types of patient‐facing ACP interventions—facilitated and patient‐directed—among 400 patients with advanced cancer and their family caregivers.^11^, ^12^ These two approaches were chosen as they are both widely used but vary in their approach and the resources required. The facilitated program involves patients (and families) receiving printed materials (a pamphlet and advance directive) and meeting with a trained nurse facilitator to have structured conversations about values, goals, and end‐of‐life decision making following the Respecting Choices First Steps Intervention model. The patient‐directed program includes PREPARE for Your Care written materials paired with an online resource that takes a step‐by‐step approach to the ACP process, which patients (and families) complete on their own time at home.^13^

In previously reported results from the parent trial, facilitated ACP resulted in greater increases in ACP engagement and advance directive completion than patient‐directed written and web‐based materials.^11^ There were no differences between groups in the odds of having a conversation about end‐of‐life wishes with family or friends or physicians, and all ACP behaviors increased significantly from baseline in both groups. As part of the larger comparative effectiveness trial, we conducted semistructured interviews with participants to learn more about factors that affected engagement with ACP for patients with cancer generally, and specifically with each approach to ACP.

## METHODS

This study was conducted as part of a larger trial comparing the effectiveness of two different approaches to ACP between 2020 and 2023. The design and primary outcomes of the larger trial have been reported separately.^11^, ^12^ The study was approved by the University of Pittsburgh Institutional Review Board (STUDY19080337). We report this study following the Consolidated Criteria for Reporting Qualitative Studies (see Supplemental Materials for COREQ checklist).^14^

The interview guide was developed using a behavioral change framework for ACP and a review of the literature on factors that impact ACP engagement.^15^, ^16^ Interview guides were iteratively edited throughout the data collection process to match the language being used by participants and to explore emerging insights. The interview guide included questions about previous engagement with ACP; experiences with ACP; and opinions about the interventions (e.g., what was helpful or not helpful). See Supplemental Materials for the Patient Interview Guide.

Inclusion criteria for the parent trial were patients aged 18 years or older, who had solid tumor malignancies, and whose oncologist “would not be surprised” if the patient died within the coming year.^11^ Additional functional and logistical criteria included an Eastern Cooperative Oncology Group score of 0 to 2 and care receipt at a participating oncology clinic We included patients who had already completed a living will or advance directive because preferences change, and patients may benefit from additional engagement in ACP. Patients were recruited from eight participating outpatient oncology clinics in western Pennsylvania.

All enrolled patients were eligible for an interview after completing the 12‐week trial outcome assessments. We aimed to sample 30 patients from each intervention arm: facilitated and patient directed. Within each intervention group, we aimed to include participants with and without a clinically significant increase in ACP engagement from baseline (defined as at least 0.2 points on the 5‐point ACP engagement scale).^17^ Consistent with the parent study design, we included patients who had completed the 12‐week assessment even if they did not engage or had low engagement with the intervention. We additionally sought to achieve maximum variation in our sampling across gender, age, and race. Enrollment was ongoing throughout the study with participants contacted by phone to ask about participating in an interview. The most common reasons for nonparticipation were no response to outreach attempts and that patients were too sick, too busy, or not interested.

One‐time interviews were conducted remotely by a trained qualitative researcher (K.R., A.R., or N.D.) who was not previously known to participants. We accommodated participants who requested that someone else (e.g., a spouse) also be present for the interview. Interviews were audio recorded, transcribed, and deidentified. Participants were compensated for their time and effort.

We used an inductive thematic approach to analysis.^18^ Members of the research team (K.R., A.R., R.I., N.D.) iteratively reviewed transcripts to discuss identified concepts and to group concepts into themes and subthemes. We continued a process of co‐coding interview transcripts to refine the thematic codebook, develop definitions, and identify inclusion and exclusion criteria for code use (see Supplemental Materials for the thematic codebook). Members of the larger research team provided input on the developing codebook during monthly meetings. This codebook development process continued until we reached thematic saturation (no new major codes were added to the codebook) and an acceptable inter‐rater reliability was achieved (kappa >0.7 and a percent agreement >95).^19^, ^20^ Next, two coders (K.R., R.I.) independently coded all the patient transcripts, meeting to review inter‐rater reliability at predetermined intervals: 25%, 50%, 75%, and 100% of the data. Data were analyzed using NVivo 13 (2020, R1) qualitative software.^21^ We reviewed themes across participants and intervention, and summarized results by themes.

## RESULTS

We interviewed 60 patients (31 patients randomized to facilitated ACP and 29 to patient‐directed ACP). Interviews averaged 25 minutes in length (range, 7 minutes 36 seconds‐1 hour 45 minutes). Participants’ mean age was 70.2 years, 53.3% were female, and 93.3% self‐reported as White. The majority were married or widowed with at least some college education. Religion played a fairly important to very important part in 81.6% of participants’ lives, with the most common religions being Catholic or Protestant. Sixty‐five percent had previously completed a living will or advance directive. More than 90% of patients interviewed were diagnosed with cancer at least 6 months prior to study inclusion. See Table 1 for participant demographics and Table 2 for participant baseline characteristics.

**TABLE 1:** Baseline demographics of interviewed patients overall and by intervention group.

Demographic	Total (*N* = 60)	Patient‐directed ACP (*N* = 29)	Facilitated ACP (*N* = 31)
Age, mean (SD), y	70.2 (10.6)	67.8(11.6)	72.5 (9.2)
Sex			
Female	32 (53.3%)	16 (55.2%)	16 (51.6%)
Male	28 (46.7%)	13 (44.8%)	15 (48.4%)
Race^a^			
African American or Black	4 (6.7%)	2 (6.9%)	2 (6.5%)
White	56 (93.3%)	27 (93.1%)	29 (93.5%)
Ethnicity^b^			
Hispanic	1 (1.7%)	0 (0%)	1 (3.2%)
Non‐Hispanic	59 (98.3%)	29 (100%)	30 (96.8%)
Educational level			
< High school	1 (1.7%)	0 (0%)	1 (3.2%)
High school diploma or GED	17 (28.3%)	9 (31.0%)	8 (25.8%)
Some college or college degree	33 (55.0%)	15 (51.7%)	18 (58.1%)
Graduate or professional degree	9 (15.0%)	5 (17.2%)	4 (12.9%)
Current marital status			
Never married	3 (5.0%)	1 (3.4%)	2 (6.5%)
Married	33 (55.0%)	19 (65.5%)	14 (45.2%)
Widowed	14 (23.3%)	4 (13.8%)	10 (32.3%)
Divorced/separated	10 (16.7%)	5 (17.2%)	5 (16.1%)
Religion			
Agnostic/atheist/no religion	4 (6.7%)	2 (6.9%)	2 (6.5%)
Catholic	21 (35.0%)	8 (27.6%)	13 (41.9%)
Other Christian	7 (11.7%)	4 (13.8%)	3 (9.7%)
Jewish	2 (3.3%)	1 (3.4%)	1 (3.2%)
Protestant	17 (28.3%)	9 (31.0%)	8 (25.8%)
Declined to answer	4 (6.7%)	2 (6.9%)	2 (6.5%)
Other	5 (8.3%)	3 (10.3%)	2 (6.5%)
**Religious importance**			
Not at all important	4 (6.7%)	2 (6.9%)	2 (6.5%)
Not too important	3 (5.0%)	2 (6.9%)	1 (3.2%)
Fairly important	14 (23.3%)	7 (24.1%)	7 (22.6%)
Very important	35 (58.3%)	16 (55.2%)	19 (61.3%)
Refused to answer	4 (6.7%)	2 (6.9%)	2 (6.5%)

*Note*: All number are n (%) except where indicated otherwise.

Abbreviations: ACP, advance care planning; SD, standard deviation.

^a^ Race and ethnicity information was self‐reported by participants.

^b^ Participants were asked to answer yes or no to the question, “Are you Latino/Latina or Hispanic or Latin American?” No additional ethnicity categories were offered.

**TABLE 2:** ACP and health history of interviewed patients overall and by intervention group.

Characteristic	Total (*N* = 60)	Patient‐directed ACP (*N* = 29)	Facilitated ACP (*N* = 31)
Confidence filling out medical forms			
Not at all	2 (3.3%)	1 (3.4%)	1 (3.2%)
A little bit	5 (8.3%)	2 (6.9%)	3 (9.7%)
Somewhat	9 (15.0%)	5 (17.2%)	4 (12.9%)
Quite a bit	15 (25.0%)	7 (24.1%)	8 (25.8%)
Extremely	29 (48.3%)	14 (48.3%)	15 (48.4%)
Cancer type			
Genitourinary	8 (13.3%)	4 (13.8%)	4 (12.9%)
Breast	13 (21.7%)	6 (20.7%)	7 (22.6%)
Gynecologic	5 (8.3%)	2 (6.9%)	3 (9.7%)
Gastrointestinal	8 (13.3%)	2 (6.9%)	6 (19.4%)
Hepatobiliary	1 (1.7%)	1 (3.4%)	0 (0%)
Head and neck	4 (6.7%)	2 (6.9%)	2 (6.5%)
Lung	8 (13.3%)	4 (13.8%)	4 (12.9%)
Pancreatic	3 (5.0%)	3 (10.3%)	0 (0%)
Prostate	9 (15.0%)	4 (13.8%)	5 (16.1%)
Other	1 (1.7%)	1 (3.4%)	0 (0%)
Patient reported previously completing a living will or advance directive	39 (65.0%)	16 (55.2%)	23 (74.2%)
Prior living will or advance directive in the medical record	9/60 (15%)	2/29 (6.9%)	7/31 (22.6%)
Prior POLST form in the medical record	1/60 (1.7%)	0/29	1/31 (3.2%)
Time since first diagnosed with cancer (at baseline)			
Less than 1 month ago	1 (1.7%)	1 (3.4%)	0 (0%)
≥ 1 month but < 6 months ago	3 (5%)	1 (3.4%)	2 (6.5%)
≥ 6 months but < 1 year ago	10 (16.7%)	7 (24.1%)	3 (9.7%)
≥ 1 year but < 2 years ago	8 (13.3%)	4 (13.8%)	4 (12.9%)
≥ 2 years but < 5 years ago	11 (18.3%)	4 (13.8%)	7 (22.6%)
≥ 5 years ago	27 (45.0%)	12 (41.4%)	15 (48.4%)

Abbreviations: ACP, advance care planning; POLST, Physician Orders for Life‐Sustaining Treatment.

### Theme 1: internal factors (attitudes, previous experiences, and motivations)

Patients discussed a broad range of internal factors that influenced their engagement with ACP, such as attitudes, previous experiences with ACP or decision making for others at the end‐of‐life, and other motivations. Quotations to support themes and subthemes are presented in Table 3.

**TABLE 3:** Themes and subthemes with quotations.

Internal Factors	
Theme and subthemes	Exemplar quotations
Attitudes ‐ realist	I’m a *realist*. I know that none of us live forever. And when you get into your 80s, and you start feeling like you’re in your 80s [Laughs]. [8036, Facilitated]
Attitudes – topic avoidance	I: What are some of the reasons you decided not to log on to the website?
	P: I just [didn’t] feel like talking about it. Or hearing about it. [8264, Patient‐directed]
	My focus has been on surviving this cancer. and I really wasn’t—it seemed like negative energy to start looking at advance care planning. [8288, Facilitated]
Previous ACP ‐ self	Well, before diagnosis, it was just something that my wife and I thought we needed to do probably on the same plane as having wills. So, in fact, that's what we did. Several years before the cancer diagnosis, we had done our wills and advanced directives. Then with the cancer diagnosis, dust it off and update it because my prior document was probably a good 10 years old or older. And so, dust it off, look at it again, make sure the directives were consistent with what we both wanted. [8858, Patient‐directed]
Previous ACP ‐ others	My mother had Alzheimer's. We were able to put things off until probably her last about 7 years. And when it became acute where my father and my husband and I could no longer safely keep her in home, then we started to sit down and talk to different caregivers and realized how much planning is needed because Mama wouldn't be able to do it by herself. So that's when we first started to realize planning is so, so important. [8749, Patient‐directed]
	Well, I knew quite a bit because I was a medical power of attorney for my aunt a long time ago, and my mother in New Jersey. And uh so I had been through the decision maker side of it in those two settings. My [pap] was in a nursing home—a veterans nursing home My mother was in a mental—uh um a dementia unit in New Jersey. And also, both of my sons suffered accidents and had very serious accidents, so we’d been through some decision making with that. And so, what‐ what [the RN] did — I thought she was fantastic — and I feel badly for people in the control arm or whatever because I thought that interviewing that [unintelligible] was really, really helpful. But she kind of, our experiences, we hadn’t really put it all together. My husband and I went, and you know, helped us kind of systematize some of the — the actions. [8131, Facilitated]
Previous ACP completed	Well it’s like I said, I’ve already drawn up the document for my self‐care plan. That’s been done. As far as I can think of at the moment, that’s all I’m going to do. [8310, Patient‐directed]
Motivations ‐ something that needs to be done	I’m just looking at it as something you need to do. You know, I have no emotions about this is all really…it’s just a matter of‐ of‐ something that I should do before something; other things I need to do also. You know? Besides just this. But it is very important that they have my directive. [8317, Facilitated]
Motivations ‐ reality of death (including cancer and age)	Well, I know there's no cure for my diagnosis, so I really had to make the decisions and wanted to make the decisions. I'm not afraid to die, and I think that that's greatly because of my faith. [9016, Facilitated]
	I just know I'm older, and it's time to think. And every once in a while, something will bring something to my mind. I just say, “What's going to be is going to be.” I have things as good as I can. It seems like through life, all you do is keep going from one—just progressing. You get married. You have children. You take care of them. The next thing, you're older. And your children are older. Here I am at the end of my life, so I've lived a long life, so that's just life. [8901, Facilitated]
Motivations ‐ alleviate burden for loved ones	Part of it made me feel I was glad I had taken the step to get these things set up for the future and wonder why‐‐ I know it's hard to think you're going to die. But to not have these I think would be very hard on, well, people like your family if you have family, if not, your friend. I mean, I wouldn't want my friend to sit here and have to go through saying, “What would she want?”and not talk to her about things like that and sort of made it easier to do that. [8778, Facilitated]
	It’s like I said, we talked to my—my sister knows, my son knows, my daughter knows. Um we talked about all this stuff and… make— to make them aware. Maybe it’ll lessen their sorrow when the time does come. [8278, Patient‐directed]
Motivations ‐ alignment with values and preferences	And plus, I'm bossy. I'm the oldest of eight. Well, I was. Now, I'm the oldest of the four of us still living. And I want control over what is done with me. When I'm not able to do it, I need to have something that says, “Here's what she wants.” And short of writing in two‐inch block letters at the bottom, “I really mean it,” I'm making an assumption that my desires will be followed at that time. [8460, Facilitated]
Motivations ‐ response to treatment	You know I guess I would have to say you know because right now my medical treatment is going pretty well and it's looking like pretty positive so I'd have to say I don't know if things were going works if I might have been you know a little more invested in getting it completed. [8366, Patient‐directed]
External factors: ACP delivery	
Theme and subthemes	Exemplar quotations
General delivery ‐ exposure	If I had the chance to participate in this study six months after my second diagnosis, I don't know if I could've done it. It took me at least that long to get my mind wrapped around what I needed to do. There is a degree of panic I think in every person if they have to face this, and your mind just goes in 20 different directions. And you have no clue where to start. And you don't want to talk about it. You don't want to think about it. You just want to clear your mind. And it takes a while to get over that. [8749, Patient‐directed]
	…like what happened with me with the study, when the opportunity presents itself and you say, oh okay here’s someone who is trained to talk about it, that you’re there or at least to act on it. Cause I don’t know whether I might have been able to decide a bit sooner if it—if I had been approached about it sooner or if I was avoiding it because the nurse brought it up before I was ready. [8424, Facilitated]
General delivery – style and design	Because it didn’t get in too heavy, but yet it got in heavy enough. You know what I mean, if you jump in too heavy, you may scare some people. [8278, Patient‐directed]
	Or not to be personal, you know when you make it personal, I think that’s when it gets to be a problem for the‐ for the patient. [8288, Facilitated]
	No, it was all pretty academic, I guess, but that's always a great benefit to the people doing the study. Not me and me. If it's animated or maybe there's a human‐interest story to read or to help make it, like I said, another way to explain it that I think that's a great benefit. Keep it light because it's all very overwhelming. [8802, Patient‐directed]
	And you could set it up—you’re healthy, but uh we thought this would be of great value to you. Um and maybe it will never come to the point that you need it. But I—I think people would be a lot more receptive to it. [8288, Facilitated]
Specific delivery – facilitated ‐ interventionist	She just has a warmth when you talk to her, and it makes you feel so comfortable you just want to tell her everything. [laughter] It was so nice to be able to let it out instead of‐‐ well, I don't want to really tell strangers. She just made it comfortable talking to her. [8778, Facilitated] So, I thought it was a very good program to ask people what they're going through. And you know more when a person explains what's happening in their life rather than just every now and then when you see a doctor, he says, “How are things?” And you just don't always give them everything. You just give a short explanation of how things are going. But this made you feel a little more comfortable that that was more in‐depth as to what you need to figure out and not shy away from it because things could get worse. [8788, Facilitated]
Specific delivery – facilitated – asking questions	And my husband had some questions that I didn’t have, or you know, there was an opportunity for everybody to discuss the same thing in the same light, in the same context. [8131, Facilitated]
Specific delivery– facilitated – having a conversation	I mean anytime—well anytime you get some more information and have a dialogue back and forth about you know what may arise or whatever is a good thing. You know, and it helps you uh, you know, maybe get a little bit clearer picture of what the… end may bring. And you know how you want to handle different things along the way. Yeah, I believe it was helpful. [8277, Facilitated]
Specific delivery – facilitated – personalized	I was just really happy that we had the personal interviews. She only had about an hour and a half, I think. But uh just the personal conversation was uh [unintelligible] was very helpful… [8131, Facilitated]
Specific delivery – patient‐directed ‐ dosing	I thought it was very well done. It was put together well. The presentation was set up so that for me, that a lot of the hard things were more towards the end than the beginning so it kind of gave you a soft start. And then it would go a little bit deeper in some places where you had to stop and walk away or think about it a little bit more. And it basically didn't throw you off the first time you opened the computer and started it. [8749, Patient‐directed]
Specific delivery– patient directed –printed materials	Yeah, we did do the paperwork first. We were sort of ready when we got to the online. Um so we sort of zipped right through that to tell you the truth. [8278, Patient‐directed]
	No. I don’t know nothing about a computer. The only thing I did was the um packet that she um you sent me. [8372, Patient‐directed]
Specific delivery– patient directed ‐videos	They were good. Um the questions that were brought up and that they played out on the videos were questions—some of them were questions that I actually had. Like, not, like the one that talks about not wanting to bother your family about doing the things that need to be done [8292, Patient‐directed]
	Not really. Like I said, watching the videos, and the videos showed all different types of points of view. But that was kind of the one that stuck with me, was how hard it can be for people to have to make decisions and not knowing exactly and hoping they're doing the right thing and the stresses that could put on them. So that was the one that really stuck out to me. I can't really think of anything else. [8381, Patient‐directed]

#### Theme 1: subtheme 1: attitudes

Some patients who self‐identified as being “realists” approached ACP with this mentality and therefore did not find it distressing to think about engaging in ACP. As one participant described, “it was just a matter of fact. I mean—all I—I’ve had different life experiences than most people. And this is just a thing you do” (8442, patient‐directed). However, some patients did not engage with the ACP interventions, were unable to overcome topic avoidance (“I try not to think about it. I'm not ready to go yet” [8660, facilitated]), or hesitated in engaging in certain ACP tasks as these were viewed as giving up hope. For example, a participant discussed not having an ACP conversation with their oncologist, “No. We haven't discussed that yet. No. I keep saying, ‘Will I be around?’ He says, ‘You're going to be around’” (8730, patient‐directed).

#### Theme 1: subtheme 2: previous experiences

Another factor that affected patients’ receptivity to ACP was having previous experience with ACP for themselves or others, including decision‐making for others. This increased participation in ACP because of familiarity with the process and/or greater recognition of its importance. As one participant noted, “My parents, when we lost them, were both way in their upper 80s, early 90s. So, we pretty much all agreed comfort is what we wanted for them. And basically, that's what I want, is comfort” (8726, facilitated). Some patients felt that they didn’t have a need to reengage with ACP as they previously documented or expressed their medical preferences. For example, one patient noted, “So I went ahead and went online and started answering questions, and then they started getting deeper into—you know, do you want this? Do you want that? And I thought, you know, I’m gonna stop right there, because I already have my wishes put on paper, and I don’t want anything else put out there that might confuse things. So I stopped” (8361, patient‐directed).

#### Theme 1: subtheme 3: motivations

Patients described several motivations for engagement. Some viewed ACP as one of many tasks that needs to be done, like checking something off a to‐do list. Others were motivated by the realization that death is an eventuality for everyone, made more pronounced by having a cancer diagnosis and, for some, their age (“I’m running out of track for this train” [8036, facilitated]). Additionally, participants wanted to alleviate burden for loved ones and lessen the worry or guilt that surrogates might have about making the correct decision. Such as, “It’s something that’s necessary to make it easier. Cause you’re always guessing otherwise what that person might want” (8278, patient‐directed). Others were motivated by having their preferences for medical care honored if they could not make decisions for themselves. As one participant stated, “The kind of cancer I got was rare, and the survival rate wasn't very good…I need to make my wishes known, so that they wouldn't do something that I didn't want them to do” (8784, patient‐directed). However, patients noted their motivation decreased when they perceived that they were responding well to their cancer treatments. As a caregiver (interviewed with a patient) described, “Now he's starting to come around a little bit. He's feeling better, gaining weight. His tests are coming back better. So you kind of slow down the [ACP] process. I'm still getting the stuff together” (8447, patient‐directed).

### Theme 2: external factors (ACP delivery generally and specifically by intervention)

#### Theme 2: subtheme 1: ACP delivery generally

Participants discussed how creating the opportunity for ACP needs to be iterative because people have varying ability and/or motivation to engage with it at different times. For example, several patients noted how they were given information about ACP right after their cancer diagnosis, and they were not emotionally capable of thinking about it at that stage.

More than 90% of patients were diagnosed with cancer at least 6 months before receiving the intervention. Participating in structured ACP created the space and opportunity for patients to engage with ACP and provided the needed resources, accessibility, and prompts. A participant described this opportunity: “Just recently, I've become a little bit more serious about this. And actually, this afternoon, I was going to go over it a little bit more with my husband…” (9152, patient‐directed).

When it came to general engagement with ACP, patients appreciated specific design features, including that the content was not too emotionally intense, that it was presented as factual, and not overly reflective of personal situations (e.g., having cancer). One participant noted, “As long as they kept it on an intellectual level and…don’t dwell on the emotional aspects then it’s fairly easy just to breeze right through it” (8044, patient‐directed). It was helpful to normalize ACP as something that everyone should do regardless of health or age. For example, “Because I would have thought, oh this is something they do for everybody. You know, not just the ones who might be dying” (8288, facilitated). Additionally, each intervention presented unique factors that impacted engagement.

#### Theme 2: subtheme 2a: factors specific to facilitated advance care planning

Patients in this group often commented on the facilitator's skills and demeanor, describing the facilitator as empathetic and knowledgeable. As one participant described, “I felt like she was coming from a position of ‘I understand, and I want to help you get past that and so we’re going to talk and then I’m going to use my magical powers and empathetic medical knowledge and I’m going to tap into something and I’m going to help you get past it’” (8424, facilitated). Some even discussed their time with the facilitator as a form of social and emotional support: “she would let you talk and let your feelings out…that made me feel comfortable…you felt at ease” (8778, facilitated). Aspects of the interaction that participants found valuable were the ability to ask questions, to have an organic conversation, and that it was personalized to the patients (and caregivers if present). One participant noted, “And the conversation, sometimes it took an unexpected turn, or you ask a question, and something came up and really something else came up as well and you’re able to address that” (8131, facilitated). Specifically, patients appreciated discussing who would be an appropriate decision maker, learning that one can give decision makers discretion (or not), and gaining a more detailed understanding of decisions related to cardiopulmonary resuscitation. Some participants met with the facilitator more than once to continue ACP conversations, ask additional questions, or include a surrogate decision maker or family member.

#### Theme 2: subtheme 2b: factors specific to patient‐directed advance care planning

Patients in this group appreciated the flexibility to self‐start, stop, and return to the patient‐directed intervention. This allowed time to think about how to respond to certain aspects as well as dose the intervention emotionally: “This way, it's kind of—I won't say impersonal—but it gives you some space…That one‐on‐one is great because you're going to get answers immediately, but a computer gives you time to walk away and regroup” (8749, patient‐directed) For some patients, reading through the printed materials was enough impetus to engage in ACP. One participant explained, “Well, I'm 67. My wife is 63. The whole website thing isn't really…we're kind of old‐school. I like looking at it on paper”(8784, patient‐directed). In addition to the written materials, others also went online and watched some of the program’s videos. Of those who did, the videos were thought to help normalize that others are going through the same thing and have the same questions, were informative about what needed to be done, and brought up different perspectives that may not have been considered. One participant noted they did not watch the videos as “they depressed me” (8379, patient‐directed). Some participants did not access the online tool because of discomfort with technology or logistical problems and, of those who did access the online tool, the majority logged on once.

## CONCLUSION

In this qualitative study examining factors that impact engagement with patient‐facing ACP, we found that for patients with cancer there were several intrinsic factors that facilitated engagement with ACP: a realist/practical approach to life that translated to an approach toward ACP, having previous experience with ACP or making decisions for others that demonstrated the value of ACP, and beliefs about consequences of doing ACP. Barriers to engagement included topic avoidance by patients and/or their social networks, reluctance to reengage if patients feel they have sufficiently completed needed activities as well as ACP not being perceived as a priority because of a positive response to cancer treatment.

Our findings align with and build on findings from a scoping review of factors that impact ACP engagement for patients with cancer.^22^ This study identified patient‐level demographic factors (e.g., race, religiosity, high death anxiety, age, level of education, lower income) as well as contextual ones (e.g., marital status, social networks, symptom burden, length of illness). Our findings align in that topic avoidance (or death anxiety) and perceptions of giving up are barriers to engagement. Although they identified that higher symptom burden decreased ACP and longer length of illness increased ACP, our participants spoke about perceptions of health in terms of the process of accepting the reality of death and response to treatment. The authors concluded that ACP should be viewed as a more dynamic process both temporally and with inclusion of available social support. Our findings similarly suggest the need to think about ACP as an ongoing process of engagement. Additionally, a systematic review of ACP for cancer patients found that patient‐directed ACP interventions allow for the discretion of involving others, emotional intensity, timing, and clinical and institutional “ownership” of the process and conversation.^23^ Our findings are similar in that participants varied in their inclusion of others and preferences for emotional intensity and timing. Interestingly, few studies in the review found that patients viewed ACP as a way to ensure patient‐centered care when they could not make decisions for themselves. However, participants in our qualitative study did express this as a specific outcome that motivated them to engage with ACP.

Although the analysis was inductive, our findings can be mapped to the domains of the COM‐B model of behavior change: capability, opportunity, and motivation.^24^ Capability encompasses the internal factors related to attitudes and previous experiences, opportunity is created by the ACP interventions of the parent trial, and motivation includes anticipated benefits for patients and families, as outlined in the results. Thinking about these findings through a COM‐B lens allows for a more nuanced understanding of the variable trajectories of ACP engagement. Not everyone will engage with the behavior, ACP, in the same way or at the same time. A constellation of internal factors act as facilitators and barriers, and the opportunity created to engage must be appropriately timed. Therefore, there is not a one‐size or one‐time‐fits‐all approach to ACP that will necessarily align with patients’ receptivity. Furthermore, as discussed by participants, the two types of interventions represent two different ways of engaging with ACP emotionally. For facilitated ACP, the interpersonal nature of the intervention led to some feeling like they could open up to the interventionist. Patient‐directed ACP is a more private and non‐interpersonal interaction that can be dosed as needed for emotional reasons. Given the differences in resource‐intensity between the two interventions, this may suggest a stepped and person‐centered approach to ACP that starts with patient‐directed printed materials and online tool, with the option to access a trained ACP facilitator for participants who require more interpersonal support.

There are several limitations to note. First, because participants were interviewed at a minimum of 12 weeks (about 3 months) following the intervention, there were difficulties in recalling the specifics of the interventions. Second, especially in early interviews, there was a learning curve as to the best language to use in asking participants interview questions. For example, few remembered the specific names of the interventions, so we adapted by asking about specific features of the experience. Third, although we attempted outreach to every patient who was non‐White for participation in an interview, our sample was not very racially diverse. The low racial diversity in the interview sample was similar to the larger study population, reflecting the demographics at recruitment clinics. Fourth, 65% of interview participants had previously completed written advance directives (compared to about 50% in the larger trial), which is higher than estimated rates among patients with advanced cancer.^25^ This may reflect a greater receptivity to ACP and over account for the role of previous experiences with ACP as a factor impacting engagement. Fifth, transcripts and findings were not reviewed by study participants. Last, our sample is limited to those who completed 12‐week study assessment. It is possible that those who did not complete the 12‐week study assessment may have provided different responses than those who did. These limitations may reduce the transferability of findings.

Our findings support developing and testing a stepped, integrated approach to both patient‐directed and facilitated ACP to accommodate different preferences for engagement.

Incorporating a longitudinal approach to ACP rather than relying on a discrete instance would better account for different stages of the cancer illness trajectory, including how motivations and ACP decisions may change over the illness trajectory. Future work should be done with more diverse patient populations, to ensure that ACP findings are generalizable to the broadest possible population of patients and their families.^26^

## AUTHOR CONTRIBUTIONS

**Kimberly J. Rak**: Conceptualization; Investigation; Writing ‐ original draft; Formal analysis; Methodology; and Data curation. **Renusha Indralingam**: Investigation; Writing ‐ review & editing; and Formal analysis. **Aaron Richardson**: Investigation; Writing ‐ review & editing; and Formal analysis. **Neha Dhole**: Investigation; Writing ‐ review & editing; and Formal analysis. **Shane Belin**: Writing ‐ review & editing; Project administration; and Data curation. **Monica C. Leon Cadavid**: Writing ‐ review & editing. **Yuxin Zhou**: Writing ‐ review & editing. **Georgeann Booth**: Writing ‐ review & editing. **Bernard Hammes**: Writing ‐ review & editing. **Betty Ferrell**: Writing ‐ review & editing. **Douglas B. White**: Writing ‐ review & editing. **Robert M. Arnold**: Writing ‐ review & editing. **Rebecca L. Sudore**: Writing ‐ review & editing. **Megan Crowley‐Matoka**: Writing ‐ review & editing; Conceptualization; and Formal analysis. **Yael Schenker**: Conceptualization; Funding acquisition; Writing ‐ review & editing; Formal analysis; and Supervision.

## CONFLICT OF INTEREST STATEMENT

Robert M. Arnold reports royalties from Cambridge University Press, UpToDate, VitaTalk. Yael Schenker reports serving as a consultant for UpToDate, Emmi Solutions. Douglas B. White reports serving in Consulting or Advisory Role for UpToDate. The remaining authors have no conflicts of interest to disclose.

## CLINICAL TRIAL REGISTRATION

This trial is registered on ClinicalTrials.gov (identifier: NCT03824158).

## PATIENT CONSENT STATEMENT

All participants were provided and signed the Institutional Review Board–approved consent form.

## Supporting information

Supplementary Material

## Data Availability

The data supporting the findings of this study are not publicly available.
