# Supplementary material for: Factors influencing engagement with patient‐directed and facilitated advance care planning interventions for patients with advanced cancer
Source: Cancer. 2025 Aug 7;131(16):e70025. doi: 10.1002/cncr.70025 (PMC12329779; doi:10.1002/cncr.70025)
Supplement: Supplementary file 1 — Supplementary Material [file CNCR-131-e70025-s001.docx]

Appendix. Factors influencing engagement with patient-directed and facilitated advance care planning interventions for patients with advanced cancer

A1. Consolidated criteria for reporting qualitative studies (COREQ): 32-item checklist

| **No. Item** | **Guide questions/description** | **Reported in** |
| --- | --- | --- |
| **Domain 1: Research team and reﬂexivity** |  |  |
| *Personal Characteristics* |  |  |
| 1. Interviewer/facilitator | Which author/s conducted the interview or focus group? | Methods |
| 2. Credentials | What were the researcher’s credentials? E.g. PhD, MD | Affiliations |
| 3. Occupation | What was their occupation at the time of the study? | Methods |
| 4. Gender | Was the researcher male or female? |  |
| 5. Experience and training | What experience or training did the researcher have? | Methods |
| *Relationship with participants* |  |  |
| 6. Relationship established | Was a relationship established prior to study commencement? | Methods |
| 7. Participant knowledge of the interviewer | What did the participants know about the researcher? e.g. personal goals, reasons for doing the research |  |
| 8. Interviewer characteristics | What characteristics were reported about the inter viewer/facilitator? e.g. Bias, assumptions, reasons and interests in the research topic |  |
| **Domain 2: study design** |  |  |
| Theoretical framework |  |  |
| 9. Methodological orientation and Theory | What methodological orientation was stated to underpin the study? e.g. grounded theory, discourse analysis, ethnography, phenomenology, content analysis |  |
| Participant selection |  |  |
| 10. Sampling | How were participants selected? e.g. purposive, convenience, consecutive, snowball | Methods |
| 11. Method of approach | How were participants approached? e.g. face-to-face, telephone, mail, email | Methods |
| 12. Sample size | How many participants were in the study? | Results |
| 13. Non-participation | How many people refused to participate or dropped out? Reasons? | Methods |
| Setting |  |  |
| 14. Setting of data collection | Where was the data collected? e.g. home, clinic, workplace | Methods |
| 15. Presence of non-participants | Was anyone else present besides the participants and researchers? | Methods |
| 16. Description of sample | What are the important characteristics of the sample? e.g. demographic data, date | Methods & Results & Tables |
| Data collection |  |  |
| 17. Interview guide | Were questions, prompts, guides provided by the authors? Was it pilot tested? | Methods, Appendix |
| 18. Repeat interviews | Were repeat inter views carried out? If yes, how many? | Methods |
| 19. Audio/visual recording | Did the research use audio or visual recording to collect the data? | Methods |
| 20. Field notes | Were ﬁeld notes made during and/or after the interview or focus group? | N/A |
| 21. Duration | What was the duration of the interviews or focus group? | Results |
| 22. Data saturation | Was data saturation discussed? | Methods |
| 23. Transcripts returned | Were transcripts returned to participants for comment and/or correction? | Discussion |
| **Domain 3: analysis and ﬁndings** |  |  |
| Data analysis |  |  |
| 24. Number of data coders | How many data coders coded the data? | Methods |
| 25. Description of the coding tree | Did authors provide a description of the coding tree? | Methods & Appendix |
| 26. Derivation of themes | Were themes identiﬁed in advance or derived from the data? | Methods |
| 27. Software | What software, if applicable, was used to manage the data? | Methods |
| 28. Participant checking | Did participants provide feedback on the ﬁndings? | Discussion |
| Reporting |  |  |
| 29. Quotations presented | Were participant quotations presented to illustrate the themes/ﬁndings? Was each quotation identiﬁed? e.g. participant number | Results & Table 3 |
| 30. Data and ﬁndings consistent | Was there consistency between the data presented and the ﬁndings? | Results, Table 3, & Discussion |
| 31. Clarity of major themes | Were major themes clearly presented in the ﬁndings? | Results |
| 32. Clarity of minor themes | Is there a description of diverse cases or discussion of minor themes? | Results |

A2. Patient Interview Guide

My name is _____.  Thank you so much for talking with us today.  I would like to ask you a few questions about your experience [meeting with Bobbi to discuss/using the PREPAREforyourCare online tool] to learn more about your values, goals, and preferences for future medical care if you were to become very sick. We want to learn more about what was helpful or not helpful about this approach to plan for future medical decisions.

There is no right answer to these questions, and your responses will be completely confidential. This interview will in no way affect your care. We can stop the interview at any point if you feel uncomfortable or do not wish to continue.  Also, feel free not to answer any question that you would prefer not to.

***ACP Definition*** *(use at any point if participant expresses confusion about what is meant by advance care planning)*


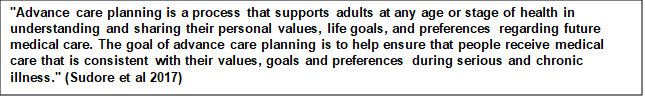


**Do you have any questions at this time? Do I have your permission to audio record our conversation?**

**[Begin recording. State the date, time, study and participant identification number]**

**First, I would like to learn a little bit about you.**

1. How would you describe your general approach to life?
2. How would you describe your approach to your cancer diagnosis?
3. What role do religious or spiritual beliefs play in your life?

**Next, I would like to talk about your experiences with planning for your future medical care if you can’t make decisions for yourself. This planning might include having discussions about your values and goals with family, with friends, and/or with your physicians.  It can also mean completing a written advance directive. With this kind of planning, the goal is to help ensure that you receive medical care that is consistent with your values, goals, and preferences during serious illness.**

1. What did you know about planning for your future medical care before this study? [probe for experiences for others]
2. How much thought did you give to this type of planning before this study?
3. Did you take any steps towards this type of planning?
4. If took steps, what was that experience like?
5. In general, what, if anything, makes it hard to do this type of planning?
6. Is there anything about your medical situation that influences how you approach this type of planning?
7. Is there anything about your religious or spiritual beliefs that influences how you approach this type of planning? Tell me more about it.
8. You are enrolled in the study with [caregiver name]. What is your relationship to them?
9. Are they your health care decision-maker? [probe for whether they have talked about it, how official it is]

**Next, I want to learn about your experience [meeting with Bobbi to discuss/using the PREPAREforyourCare** **online tool to learn more about] your values, goals, and preferences regarding future medical care.**

1. **[For PREPARE]** Did you read any of the printed materials that were sent to you as part of the study?

**If yes,** were these helpful in any way?

1. Did you use the printed materials?
2. If so, how did you use them?
3. Did you log on to the website?
4. **If not logged on**, what were some reasons you did not log on?
5. **If logged on,** did anyone else participate with you? [probe for who]

- How many times did you log on and use the website?
- Were you able to complete all five sections?
- If not, what were the reasons you were not able to complete all the sections?
- What did you think about how the site was organized?
- Did you watch the videos?
- If yes, were these helpful in any way?

1. **[For Respecting Choices**^®^**]** How did you meet with Bobbi—by phone, video, or in-person?
2. What was the reason you chose to meet by [phone/video/in-person]?
3. Was there anything that you liked or did not like about meeting by [phone/video/in-person]?
4. Did anyone else participate with you?
5. Was there anything that you particularly liked about your experience [meeting with Bobbi/using the PREPAREforyourCare online tool]?
6. Was there anything that you didn’t like about it?
7. Were there parts of this experience that were harder to do?  Tell me more about that.
8. Is there anything that you can think of that would have made it easier for you to participate in [the conversations with Bobbi/use the PREPAREforyourcare online tool]?

**Next, I would like to learn more about what was or was not helpful about [the times you met with Bobbi/used the printed materials and online tool].**

1. How did [those conversations with Bobbi/the printed materials and using the online tool] make you feel?
2. Is there anything about [talking with Bobbi/ the printed materials and online tool] that was especially useful for you?
3. Was there anything that was not useful about it?
4. Are there any ways [having those conversations with Bobbi/ using study materials and the website] made it easier to take next steps in planning for future medical care?
5. Is there anything you learned from participating?
6. Were you able to use any of the things you learned?

        16. Did [talking with Bobbi/ the printed materials and using the online tool] lead to any conversations with family about what is important to you regarding your values, goals, and preferences for future medical care? Tell me more about that.

1. If conversations took place: was [talking with Bobbi/ using the online tool] helpful in having conversations with family? (e.g. gain any new skills)
2. If no conversations took place: tell me more about the reasons you have not talked with family.

        17. What is your relationship like with your physicians?

1. Did [talking with Bobbi/ the printed materials and using the online tool] lead to any conversations with physicians about what is important to you regarding your values, goals, and preferences for future medical care? Tell me more about that.
2. If conversations took place, was [talking with Bobbi/the printed materials and using the online tool] helpful in having conversations with physicians? (e.g. gain any new skills)
3. If no conversations took place, tell me more about the reasons you have not talked with your physicians.

        18. Have you had conversations with any other members of your care team? Probe for who and what discussed.

        19. Did [talking with Bobbi/ the printed materials and online tool] lead to writing down any preferences you have for future medical care (for example, in an advance directive)? Tell me more about that.

1. If written down, was [talking with Bobbi/ the printed materials and online tool] helpful in writing down your preferences?  (e.g. gain any new skills)
2. **For PREPARE**, did you use the paper document given to you by the study?
3. If not written down, tell me more about the reasons you have not written down your preferences.

**To finish,**

       20. What was your opinion of [meeting with Bobbi/ using printed materials and the online tool] to think about planning for future medical decisions?

       21. Would you recommend [meeting with Bobbi/ using the printed materials and the online tool] to other patients and families? Probe for reasons.

       22. If you could make any recommendation to improve your experience, what would that be?

       23. Do you feel you have done what you need to do at this point to prepare for future decision making, or are there still things you need to do? Please tell me more about that.

       24. Is there anything else that you think we should know about your experience?

**[Stop recorder]**

**Thank you so much for your time.** Do you have any questions for me?

A3. Patient Thematic Codebook

| **Codes** |  |  | |  | **Definitions** |
| --- | --- | --- | --- | --- | --- |
| Intervention participation |  | |  |  | Referring to facilitated or patient-directed intervention participation |
|  | Patient |  | |  | Referring to facilitated or patient-directed interventions from perspective of patient |
|  |  | Where | |  | Reference to place or position the intervention took place |
|  |  | With whom | |  | Includes references to individuals who accompanied patient during intervention |
|  |  | Times interacted | |  | Number of times patient participated in intervention either in-person or online |
|  |  | Engagement | |  | Depth/level of engagement with intervention (ex. not watching all videos in patient-directed) |
|  |  |  | | Partial | Indications that patient started intervention but did not complete the intervention in its entirety |
|  | CG |  | |  | Referring to facilitated or patient-directed interventions from perspective of caregiver |
|  |  | Where | |  | Reference to place or position the intervention took place |
|  |  | Engagement | |  | Depth/level of engagement with intervention (ex. not watching all videos in patient-directed) |
|  |  |  | | Partial | Indications that patient started intervention but did not complete the intervention in its entirety |
| Stages of ACP |  |  | |  | Which stages of ACP achieved by participant only for after intervention |
|  | Talked |  | |  | Information or feelings expressed by patient in reference to EOL wishes or ACP |
|  |  | SDM | |  | Whether or not had conversation(s) with surrogate decision maker |
|  |  | Caregiver | |  | Whether or not had conversation(s) with caregiver if different from SDM |
|  |  | Family | |  | Whether or not had conversation(s) with other family members |
|  |  | Medical Team | |  | Whether or not had conversation(s) with members of patient's medical team (broadly or cancer specific) |
|  |  | Other | |  | Whether or not had conversation(s) with others |
|  | Has SDM |  | |  | Patient has selected a surrogate decision maker (SDM); includes verbal agreement between patient and SDM |
|  |  | Changed SDM | |  | Patient has changed who their SDM is as a result of realizing someone else is better able to act in that role. Co-code with Intervention Attributes if this is a result of the intervention. |
|  | Decided on choices |  | |  | Patient has made decision on EOL wishes and may or may not have written them down and may or may not be on record |
|  |  | May change in the future | |  | While patient may have decided on choices they recognize that their decisions may change |
|  | Written |  | |  | Patient has written advanced directive, but it may or may not be finalized or witnessed |
|  |  | Witnessed | |  | Patient has written advanced directive that has been witnessed and signed |
|  |  | Not witnessed | |  | Patient has written advanced directive but has not been witnessed and signed |
|  |  | Update previous | |  | When participation led to updating an existing living will/AD |
|  | On record |  | |  | The witnessed advanced directive is in the medical record or file. |
|  | Back up person |  | |  | Patient has identified additional person to serve as SDM |
|  | Relative stage |  | |  | Participants perception of whether there are still things they need to do related to ACP or that they have done what they want |
|  |  | Sufficient | |  | Participants have done what they feel they want to do related to ACP |
|  |  | Planning to | |  | Patient plans to complete any of the stages of ACP in the future (talking, selecting an SDM, writing down decisions, witnessing, or submitting advanced directive, etc.) *Co-code with relevant codes* |
| Psychological result of ACP |  |  | |  | How patients, caregivers, and others feel after doing ACP/that ACP is done |
| Health status |  |  | |  | References to how current health status affects other codes |
|  | Age |  | |  | References to how age affects other codes |
|  | Diagnosis |  | |  | References to how the cancer diagnosis factored into ACP |
| Spirituality |  |  | |  | Ways in which spiritual beliefs of patients and caregivers affect approach to ACP |
| Relationship with Medical Team |  |  | |  | Discussions of the evaluative and interpersonal components of the relationship between the patient/family and the patient's medical team |
| Previous experiences with ACP |  |  | |  | References to experiences with ACP before intervention |
|  | Personal |  | |  | Previous experiences with ACP before intervention |
|  |  | Talked | |  | Information or feelings expressed by patient in reference to EOL wishes or ACP |
|  |  |  | | SDM | Conversation(s) with surrogate decision maker |
|  |  |  | | Caregiver | Conversation(s) with caregiver if different from SDM |
|  |  |  | | Family | Conversation(s) with other family members |
|  |  |  | | Medical Team | Conversation(s) with members of patient's medical team (broadly or cancer specific) |
|  |  |  | | Other | Conversation(s) with others |
|  |  | Has SDM | |  | Patient has selected a surrogate decision maker (SDM); includes verbal agreement between patient and SDM |
|  |  | Decided on choices | |  | Patient has made decision on EOL wishes but has not written them down |
|  |  | Written | |  | Patient has written advanced directive may or may not be finalized or witnessed |
|  |  |  | | Witnessed | Patient had written advanced directive that had been witnessed and signed |
|  |  |  | | Not witnessed | Patient had written advanced directive but had not been witnessed and signed |
|  |  | On record | |  | The witnessed advanced directive is in the medical record or file. |
|  |  | Back up person | |  | Patient has identified additional personnel to serve as SDM |
|  | For others |  | |  | Patient has experiences with ACP through another individual (i.e. family/friend) |
| Social network |  |  | |  | The people in the patient's life that they can turn to for support, broadly speaking. |
|  | Family |  | |  | Family as support network |
|  | Friends |  | |  | Friends as support network |
|  | Other |  | |  | Support outside family and friends (e.g. support groups, church) |
| Identity attributes |  |  | |  | **General** approaches to life or deeply rooted personal principles |
|  | Coping strategies |  | |  | Ways patients describe managing or processing living with cancer |
|  | Approach to ACP |  | |  | Descriptions of the psychological or emotional framing in approach to ACP |
| Intervention specific attributes |  |  | |  | Things liked and not liked about interventions as well as suggested changes to improve. Also, notion of value related to if this is something that would benefit other patients and families. |
|  | Motivation to participate |  | |  | Factors that impact a patient's motivation to participate/engage with intervention |
|  | Opinion of OL |  | |  | Likes and dislikes of patient-directed |
|  |  | Videos | |  | Likes and dislikes of patient-directed videos |
|  |  | Difficult sections | |  | Areas or sections of ACP intervention that are identified as harder to do |
|  |  | Helpful | |  | Aspects of intervention patient identified as being beneficial or useful |
|  |  |  | | Reassured | Descriptions by patients and caregivers who have previously done ACP that it confirmed they were on the right track or confirmed that they did what they needed to do. Or future oriented that participants are reassured that what they are planning to do is appropriate. |
|  |  |  | | Learned | Knowledge that patient acquired as a direct result of intervention; Includes information wished learned |
|  |  | Not helpful | |  | Aspects of intervention patient identified as being not beneficial or useful |
|  |  | Feeling | |  | An emotional state or reaction towards/during the intervention |
|  | Opinion of facilitated |  | |  | Likes and dislikes of facilitated |
|  |  | Asking questions | |  | Ability, and the likes and dislikes, of having a dialogue with the interventionist facilitating facilitated |
|  |  | Interventionist | |  | Likes and dislikes of interventionist facilitating facilitated |
|  |  |  | | Social Support | References to how patients may or may not turn to facilitated facilitator for emotional support |
|  |  | Personalized | |  | Expression of how the facilitated session(s) were tailored to patients (not overly generalized) |
|  |  | Difficult sections | |  | Areas or sections of ACP intervention that are identified as harder to do |
|  |  | Helpful | |  | Aspects of intervention patient identified as being beneficial or useful |
|  |  |  | | Reassured | Descriptions by patients and caregivers who have previously done ACP that it confirmed they were on the right track or confirmed that they did what they needed to do. Or future oriented that participants are reassured that what they are planning to do is appropriate. |
|  |  |  | | Learned | Knowledge that patient acquired as a direct result of intervention; Includes information wished learned |
|  |  | Not helpful | |  | Aspects of intervention patient identified as being not beneficial or useful |
|  |  | Feeling | |  | An emotional state or reaction towards/during the intervention |
|  | Impact on previous ACP |  | |  | How did having previously done ACP (written but sometimes decided on choices) interacts with thoughts and opinions about the interventions |
|  |  | Added value | |  | How having done ACP previously, participants still find value in the intervention |
|  |  | No added value | |  | How having done ACP previously, participants do not find value in the intervention |
|  | Recommendations |  | |  | General recommendations on how to improve both intervention types |
|  | Recommend to others |  | |  | Whether (and to whom) would recommend utilizing ACP intervention and why. |
|  | Opinion of alternative ACP delivery |  | |  | Expression of relative value if they had received the other intervention versus the one they did receive |
|  | Printed materials |  | |  | References to informational printed materials given to participants in patient-directed intervention |
| Barriers to ACP |  |  | |  | Reasons why someone did not or might not do ACP |
|  | Logistical/access issues |  | |  | Practical aspects that act as a barrier towards engagement with the intervention |
|  | Opportunity created |  | |  | Lack of externally created space to promote/encourage discussions about ACP |
|  | Reasons for not doing ACP |  | |  | Reasons for not doing ACP |
|  | Timing |  | |  | Description of how the timing of the receipt of the intervention was a negative (e.g., too early or too late) |
|  | Topic avoidance |  | |  | Not wanting to think/talk about death/dying |
|  |  | Reluctance to start conversation | |  | When the patient or caregiver discuss not wanting to be the one to initiate the conversation. A sense of we will talk about it when (the other) is ready to |
|  |  | Patient | |  | When the patient describes not wanting to think/talk about death/dying, as well as others describing patient not wanting to think/talk about death/dying |
|  |  | Caregiver | |  | When the caregiver describes not wanting to think/talk about death/dying, as well as others describing caregiver not wanting to think/talk about death/dying |
|  |  | SDM | |  | When the SDM describes not wanting to think/talk about death/dying, as well as others describing the SDM not wanting to think/talk about death/dying |
|  |  | Other | |  | When other family and friends do not want to think/talk about death/dying, as well as others describing them not wanting to think/talk about death/dying |
|  | Maintaining flexibility |  | |  | Not wanting to write down preferences (or if written give them to their medical team) as there are things that may change or they are worried that it will be too rigid |
|  | Privacy |  | |  | Feeling that the topic or content is sensitive and wanting to limit involvement of others |
|  |  | Security | |  | Concerns about personal information (e.g. online) |
| Facilitators to ACP |  |  | |  | Reason to do or makes it easier to do ACP (or why they did do it) for both patient and cg |
|  | Empowerment |  | |  | Discussion of translating how something learned led to the ability to enact/engage with an aspect of ACP as well as increased confidence that they can or will engage with an aspect of ACP |
|  | Logistical/access issues |  | |  | Practical aspects that facilitate engagement with the intervention |
|  | Motivation to do ACP |  | |  | Factors that motivate patient to engage with ACP, as described by patients, caregivers, and physicians |
|  |  | Something that needs to be done | |  | ACP is described as something that should be done/taken care of (sometimes framed as similar to other things in life like insurance, or having a will) |
|  |  | Reality of death | |  | Recognition of the eventuality of death and how that serves as a motivator to complete ACP |
|  |  | Help others | |  | Discussion of how doing ACP could benefit friends and family in making decisions and processing their death |
|  |  | Patient well-being | |  | Discussion of how alignment of patient's wishes or ensuring patient centered care motivates them to complete ACP |
|  | Opportunity created |  | |  | Externally created space to promote/encourage discussions and/or engagement about ACP. Including how the timing of the receipt of the intervention was a motivator. |
|  | Style/type of engagement |  | |  | Ways participants describe it is easiest or better to engage with ACP (e.g. keep on an intellectual level) |
|  |  | Intellectualization | |  | A clinical framing of the material that downplays emotions. Includes hypotheticals that could happen to anyone rather than too close to personal health status (e.g. cancer). |
|  |  | Normalize | |  | When ACP is framed/viewed as a routine task for everyone regardless of age and health status (It is something anyone can or should do) |
|  |  | Direct and In-depth | |  | When ACP is framed/viewed as something that enables participants to be open and detailed about their conditions, needs, and feelings. |
|  |  | Intensity | |  | Discussions of how the content of the interventions were emotionally acceptable (not too heavy or deep) including the ability to "dose" the intervention to address emotional impacts *****6/6/24: 8749pt: idea that you can step away from intervention if too emotionally intense** |
| Belief that choices will be followed |  |  | |  | Both alignment and fulfillment of choices with SDM but also social network |
|  | Uncertain that choices will be followed |  | |  | Belief that SDM (or others) may not follow through with the patient's wishes (e.g., want to do everything possible) |
| Assumed to know |  |  | |  | When either patients or caregivers allude to a generalized, potentially undiscussed, "knowing" of what to do even when there is an absence of discussion that decisions have been made |
| Decision discretion |  |  | |  | Discussions related to how much leeway to allow decision makers or ability to change facets of existing ACP (situational considerations e.g., allow for care if expectation is the patient would survive). Caregivers and/or patients expressing flexibility about areas of ACP that potentially have more ambiguity or personalized options (e.g. the idea of time-limited trials) |
| SDM Role |  |  | |  | How either the patient, the SDM, or HCP describe the role and responsibilities of SDM |
